# Transforming growth factor-β1 polymorphisms and graft-versus-host disease risk: a meta-analysis

**DOI:** 10.18632/oncotarget.6289

**Published:** 2015-11-03

**Authors:** Lin Zhang, Lihong Mao, Junxiu Xu

**Affiliations:** ^1^ Department of Clinical Laboratory, The Fifth Affiliated Hospital of Zhengzhou University, Zhengzhou, Henan, China

**Keywords:** graft-versus-host disease, TGF-β1, meta-analysis, genetic

## Abstract

Some studies have demonstrated that transforming growth factor (TGF)-β polymorphisms may have an important role in the pathological process of graft-versus-host disease (GVHD). However, the results are not consistent. Thus, we performed a meta-analysis. Online databases were searched to obtain relevant articles published up until May 2015. Odds ratios (ORs) with 95% confidence intervals (CIs) were used to assess the strength of associations. Donors (OR=0.56; 95%CI, 0.32–0.98; P=0.04) and recipients (OR=0.73; 95%CI, 0.63–0.85; P<0.0001) with TGF-β1 rs1800469 polymorphism showed decreased GVHD risk, respectively. Donors with TGF-β1 rs1800470 polymorphism were also observed to have lower GVHD risk (OR=0.65; 95%CI, 0.46–0.94; P=0.02). However, TGF-β1 rs1800470 polymorphism in recipients was not associated with GVHD risk (OR=1.28; 95%CI, 0.81–2.01; P=0.29). No significant heterogeneity was found in the meta-analysis. This meta-analysis suggests that donors or recipients with TGF-β1 rs1800469 polymorphism and donors with TGF-β1 rs1800470 polymorphism might be associated with reduced GVHD risk.

## INTRODUCTION

Graft-versus-host disease (GVHD) is an immune driven disorder where donor T cells react and proliferate in response to host antigens [1]. GVHD remains one of the leading causes of morbidity and mortality associated with allogeneic transplantation in patients and stands as a significant barrier to the broader use of hematopoietic stem cell transplantation (HSCT) [2]. The development of GVHD appears to be determined by genetic and environmental factors. Many genes have been shown to be involved in its pathogenesis [3].

Transforming growth factor (TGF)-β is a multiplicity factor mediating cellular processes, including cell growth, cell differentiation, apoptosis, and cellular homeostasis [4]. Recent studies have proposed a role for TGF-β in controlling T-cell alloreactivity and have confirmed that G-CSF administration to human stem-cell donors results in an increase in TGF-β from CD4 T cells [6]. This is also consistent with the clinical association of reduced serum TGF-β levels after engraftment and severe GVHD [6]. Additionally, Li et al. showed that a significant decrease in the levels of TGF-β was seen with increased severity of GVHD [7].

Some studies have investigated the associations between the TGF-β1 polymorphisms and susceptibility of GVHD [8-15]. Most of the studies focused on two polymorphisms: +869C/T (rs1800470) and −509C/T (rs1800469). However, the associations observed between two polymorphisms and the risk for GVHD were controversial and inconclusive. This meta-analysis aimed to comprehensively explore the associations between the TGF-β1 polymorphisms and risk of GVHD.

## RESULTS

### Study characteristics

The baseline characteristics of the literatures enrolled were summarized in Table 1. Nine studies were included in this meta-analysis. All studies were published between 2001 and 2014. Of these, the majority of the studies were executed in Caucasian (n = 7). Others were conducted in Asian (n = 2). The total sample size from all studies was 1651 and the sample size was 20–394 patients and the range of medium age was 22–48 years. Two studies reported the associations between TGF-β1 polymorphisms and GVHD severity, while other studies reported the associations between TGF-β1 polymorphisms and GVHD risk. All studies were assessed by NOS. The quality scores ranged from 6 to 8, suggesting that the methodological quality was acceptable.

**Table 1:** Characteristics of the studies included in this meta-analysis

Study	Year	Ethnicity	Age	No. of Subjects	No. of GVHD (+)	No. of GVHD (−)	HLA matched	GVHD grade	Quality assessment
Holweg	2001	Caucasian	47	236	72	164	Mixed	NA	7
Visentainer	2005	Caucasian	30	118	19	99	Yes	Mixed	7
Noori-Daloii	2007	Caucasian	25	84	42	42	Yes	NA	8
Shah	2009	Caucasian	42	46	46	0	Yes	Reported	7
Berro	2010	Caucasian	NA	427	117	310	Mixed	Mixed	8
Karimi	2010	Caucasian	NA	20	20	0	NA	Reported	6
Rashidi–Nezhad	2010	Caucasian	22	86	43	43	Yes	NA	7
Xiao 1	2010	Asian	24	138	94	44	Mixed	Mixed	8
Xiao 2	2010	Asian	32	102	24	78	Yes	NA	8
Kim	2014	Asian	48	394	307	87	Mixed	NA	7

GVHD, acute graft versus host disease; HLA, human leukocyte antigen; NA, not available.

### Quantitative Data Synthesis

### TGF-β1 polymorphisms and GVHD risk in donors

**Figure 1:**
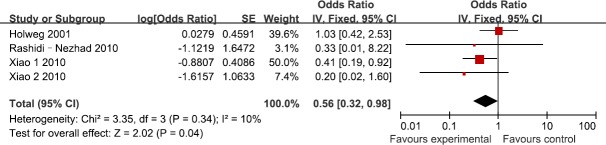
Meta-analysis for for TGF-β1 rs1800469 polymorphism and GVHD risk in donors

Four studies reported the association between TGF-β1 rs1800469 polymorphism and the susceptibility to GVHD in donors. According to the heterogeneity test, the studies showed no significant heterogeneity (I^2^=10%, P=0.34). Our findings demonstrated that TGF-β1 rs1800469 polymorphism reduced the susceptibility to GVHD (OR=0.56; 95%CI, 0.32–0.98; P=0.04; Figure 1). In the subgroup analyses by ethnicity, the significant association was only found among Asians (OR=0.38; 95%CI, 0.18–0.80; P=0.01), while no significant association was found among Caucasians (OR=0.95; 95%CI, 0.40–2.25; P=0.12). In addition, no significant association was found among HLA-matched subjects (OR=0.23; 95%CI, 0.04–1.32; P=0.10).

**Figure 2:**
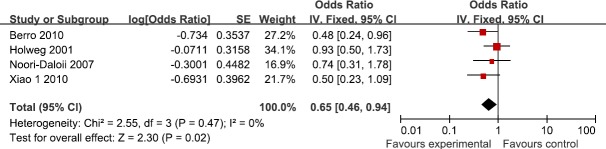
Meta-analysis for for TGF-β1 rs1800470 polymorphism and GVHD risk in donors

Four studies reported the association between TGF-β1 rs1800470 polymorphism and the susceptibility to GVHD in donors. According to the heterogeneity test, the studies showed no heterogeneity (I^2^=0%, P=0.47). Our findings demonstrated that TGF-β1 rs1800470 polymorphism reduced the susceptibility to GVHD (OR=0.65; 95%CI, 0.46–0.94; P=0.02; Figure 2). In the subgroup analyses by ethnicity, no significant association was found among Caucasians (OR=0.70; 95%CI, 0.47–1.06; P=0.09). All the results are listed in Table 2.

**Table 2:** Results of the meta-analysis

Polymorphisms	Subgroup	Heterogeneity		Test of association		
		OR (95% CI)	*P* Value	Model	I2 (%)	*P* Value
Donor						
rs1800469	Overall	0.56 (0.32-0.98)	0.04	F	10	0.34
	Caucasian	0.95 (0.40-2.25)	0.12	F	0	0.50
	Asian	0.38 (0.18-0.80)	0.01	F	0	0.52
	HLA-matched	0.23 (0.04-1.32)	0.10	F	0	0.80
rs1800470	Overall	0.65 (0.46-0.94)	0.02	F	0	0.47
	Caucasian	0.70 (0.47-1.06)	0.09	F	0	0.37
Recipient						
rs1800469	Overall	0.73 (0.63-0.85)	<0.0001	F	7	0.37
	Caucasian	0.51 (0.22-1.18)	0.12	F	0	0.78
	Asian	0.74 (0.63-0.86)	0.0001	F	42	0.18
	HLA-matched	0.32 (0.08-1.27)	0.11	F	0	0.99
rs1800470	Overall	1.28 (0.81-2.01)	0.29	F	0	0.72
	Caucasian	1.32 (0.80-2.17)	0.28	F	0	0.44

F, fixed effects model; HLA, human leukocyte antigen.

### TGF-β1 polymorphisms and GVHD risk in recipients

**Figure 3:**
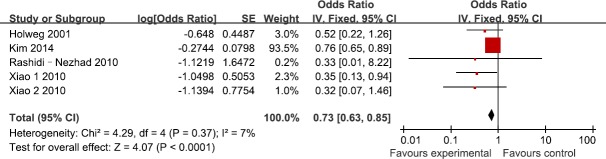
Meta-analysis for for TGF-β1 rs1800469 polymorphism and GVHD risk in recipients

Five studies reported the association between TGF-β1 rs1800469 polymorphism and the susceptibility to GVHD in recipients. According to the heterogeneity test, the studies showed no significant heterogeneity (I^2^=7%, P=0.37). Our findings demonstrated that TGF-β1 rs1800469 polymorphism reduced the susceptibility to GVHD (OR=0.73; 95%CI, 0.63–0.85; P<0.0001; Figure 3). In the subgroup analyses by ethnicity, the significant association was only found among Asians (OR=0.74; 95%CI, 0.63–0.86; P=0.0001), while no significant association was found among Caucasians (OR=0.51; 95%CI, 0.22–1.18; P=0.12). In addition, no significant association was found among HLA-matched subjects (OR=0.32; 95%CI, 0.08–1.27; P=0.11).

**Figure 4:**
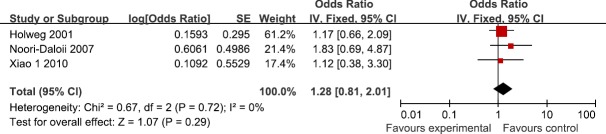
Meta-analysis for for TGF-β1 rs1800470 polymorphism and GVHD risk in recipients

Three studies reported the association between TGF-β1 rs1800470 polymorphism and the susceptibility to GVHD in recipients. According to the heterogeneity test, the studies showed no heterogeneity (I^2^=0%, P=0.72). Our findings demonstrated that TGF-β1 rs1800470 polymorphism did not influence the susceptibility to GVHD (OR=1.28; 95%CI, 0.81–2.01; P=0.29; Figure 4). In the subgroup analyses by ethnicity, no significant association was found among Caucasians (OR=1.32; 95%CI, 0.80–2.17; P=0.28). All the results are listed in Table 2.

## DISCUSSION

TGF-β can inhibit TH1 and TH2 differentiation and the acquisition of most, if not all, effector functions by naive T cells [16]. During GVHD, systemic levels of IFN-γ were increased in the presence of TGF-β neutralization, suggesting the augmentation of type 1 differentiation. Acute GVHD has been established as a T-cell-dependent and a TH1-dominant disease [17], and the enhancement of T-cell proliferation or TH1 differentiation because of a reduction in regulation appears the predominant mechanism by which the neutralization of TGF-β augments the severity of GVHD. TGF-β1 rs1800470 and rs1800469 polymorphisms have been identified. These allelic variants were associated with increased level of TGF-β1 expression and plasma concentration [18, 19]. Therefore, it is possible that TGF-β1 rs1800470 and rs1800469 polymorphisms could decrease the risk of GVHD.

This meta-analysis assessed the association between TGF-β1 rs1800470 and rs1800469 polymorphisms and GVHD risk systematically. Donors or recipients with TGF-β1 rs1800469 polymorphism showed decreased GVHD risk. Donors with TGF-β1 rs1800470 polymorphism were also observed to have lower GVHD risk. However, TGF-β1 rs1800470 polymorphism in recipients were not associated with GVHD risk. There was only three studies investigated the association between TGF-β1 rs1800470 polymorphism and GVHD risk. Thus, more studies are needed to determine whether recipients with TGF-β1 rs1800470 polymorphism have lower GVHD risk. Only two studies evaluated the association between TGF-β1 polymorphisms and GVHD severity. Thus, meta-analysis was not conducted. Furthermore, all these two studies did not find significant association TGF-β1 polymorphisms and GVHD severity.

Limitations of the present study should be acknowledged. First, the sample sizes in several of the incorporated studies were relatively small, which may reduce the strength of our conclusions. Second, all eligible studies were published in English and indexed by the selected databases. It is possible that studies published in other languages or unpublished studies could be missed, which might bias the results. In addition, the result was on the basis of unadjusted estimates, while a more accurate analysis should be carried out if more detailed individual information was available, which would allow for an adjusted estimate by other factors.

In summary, despite the limitations, this meta-analysis suggests that donors or recipients with TGF-β1 rs1800469 polymorphism and donors with TGF-β1 rs1800470 polymorphism might be associated with reduced GVHD risk. More future studies with good methodology design are warranted.

## MATERIALS AND METHODS

### Publication search

PubMed, EMBASE, Web of Science, and the Cochrane Library were searched to obtain relevant articles published up until May 2015. The following medical subject heading (Mesh) terms were used in combination with Boolean operators AND or OR: graft-versus-host disease, GVHD, Transforming growth factor, TGF, and TGF-β1. Furthermore, the references in relevant articles were screened manually to identify additional eligible studies. No language restriction was imposed during the electronic search.

### Inclusion and exclusion criteria

**Figure 5:**
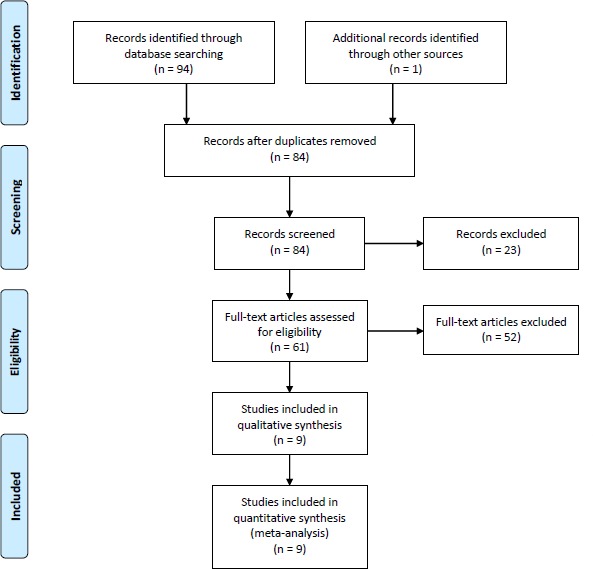
Flow chart for the literature search strategy

We selected eligible studies based on the following criteria: (1) cohort or case-control study; (2) investigated associations between TGF-β1 rs1800470 or rs1800469 polymorphism and GVHD; (3) provided sufficient data of allele and genotype frequencies of SNPs or required information could be calculated; (4) if serial studies on the same population were published, only the most recent study was included; (5) proper methodology design. Two independent investigators performed study selection and reached final consensus. The details of literature search and selection were shown in Figure 5 in standard PRISMA flow diagram style.

### Qualitative assessment

Two authors completed the quality assessment independently. The Newcastle–Ottawa Scale (NOS) was used to evaluate the methodological quality, which scored studies by the selection of the study groups, the comparability of the groups, and the ascertainment of the outcome of interest. We considered a study awarded 0-3, 4-6, or 7-9 as a low-, moderate-, or high-quality study, respectively [20].

### Data extraction

The data were extracted from each included study by two independent investigators, and the following information was collected: surname of the first author, year of publication, ethnicity, age, sample size, human leukocyte antigen (HLA) status, and GVHD grade. Disagreement on the inclusion of any study was resolved by consultation with a third investigator.

### Statistical analysis

The associations between TGF-β1 polymorphisms and GVHD risk was assessed using odds ratios (ORs) and the corresponding 95% confidence intervals (CIs) in donors and recipients, respectively. Dominant model was used in this meta-analysis, because most of the studies reported the results in this model. The heterogeneity of the included trials was assessed by the Cochrane's Q statistic for each meta-analysis. We carried out both fixed-effects (Mantel–Haenszel method) and random effects (DerSimonian–Laird method) models and producted the pooled HRs. In addition, subgroup analyses were performed to investigate the potential causes of heterogeneity according to ethnicity and HLA status. Publication bias was evaluated if more than ten studies were included. All analyses were performed by using stata 12.0 statistical software (Stata Corporation, College Station, TX, USA). All *P* values were two-sided.
